# Systematic librarian-led zip code analysis to target underserved communities

**DOI:** 10.5195/jmla.2025.2053

**Published:** 2025-07-01

**Authors:** Rachel Roberts, Kelsey L. Grabeel

**Affiliations:** 1 reroberts@utmck.edu, Assistant Professor, Preston Medical Library / Health Information Center, University of Tennessee Graduate School of Medicine / University of Tennessee Medical Center, Knoxville, TN; 2 KGrabeel@utmck.edu, Professor, Preston Medical Library / Health Information Center, University of Tennessee Graduate School of Medicine / University of Tennessee Medical Center, Knoxville, TN

**Keywords:** Health literacy, outreach, consumer library, hospital librarianship, Social determinants of health, Hospital

## Abstract

**Background:**

To compare the library's health information service usage area and customer topics with the hospital's reasons for hospitalizations to examine commonalities and explore potential growth opportunities within the community.

**Case Presentation:**

Researchers partnered with the hospital for this project. IRB approval was received. Researchers gathered the health information service's 2022 data, which was de-identified. Data analyzed included zip code and customer topics, which were coded according to the hospital's business line, which was defined as why a patient was hospitalized or used the ED. The health information service's business lines were compared with the hospital's business lines. Lastly, researchers also reviewed the hospital's targeted zip codes to see if those overlapped with the top zip codes that utilize the health information service. The top zip codes that used the library's health information service were 37920, 37918, 37917, 37919, and 37876. Usage of the health information service varied across zip codes and topics. The most requested topics for the health information service and reasons for hospitalizations/ED visits were General Medicine in three of the five zip codes. Based on the data's results, librarians performed outreach to organizations in the targeted zip codes to increase visibility of the library's services.

**Conclusion:**

The reasons people requested health information from the library aligned with hospitalizations and ED visits in most of the zip codes. Providing further outreach to the hospital's targeted zip codes will benefit both the hospital and the library by increasing usage of the health information service.

## BACKGROUND

Throughout the years, hospitals have provided the community with medical and surgical care. According to the American Hospital Association, in 2022, there were over 6,000 hospitals in the United States (U.S.) and over 33 million admissions [1]. In 2021, there were over 139 million Emergency Room (ER) visits [3]. With so many hospitalizations in the U.S., there is no wonder why 72% of adult internet users have searched online for health information [4]. Although many people may turn to Google to find health information, this can lead to misinformation. Additionally, many studies have found that online patient education is written at a grade level higher than the average reading grade level [5]. By having a hospital library, physicians have a place to send their patients to find reliable and accurate health information instead of telling them to find the information online [6]. Many studies have found that the library and its resources have a positive impact on patient care [7]. Having a dedicated patient and family library has been shown to improve the patient and family experience [8].

While all libraries that provide health information services are essential to promoting access to healthcare and health information, this is especially true in rural communities that lack resources like Internet access and access to reliable health information [11]. Hospital libraries are also valuable for communities that lack resources like Internet access. These populations are more likely to call or come in person for health information, so the librarians can provide grade level appropriate information following a reference interview in which they evaluate a patient's health literacy level, which refers to a person's ability to understand health information and use that information to make health-related decisions [9, 10]. Hospital libraries positively contribute to a person's health literacy, which is a defining aspect of overall community health [12].

Libraries often partner with community organizations to raise awareness of library services and target specific needs [11]. While public libraries receive questions about health topics on a regular basis, not all public librarians feel comfortable answering health related questions [13]. This makes hospital libraries a crucial and unique service for communities. However, a service is only beneficial if people know about it. Raising awareness for an unknown service requires an intentional marketing plan. Direct marketing, such as sending brochures to various organizations in the community, is a proven way to increase patron awareness of a service [14].

The University of Tennessee Medical Center (UTMC), a teaching hospital located in the middle of Knox County, TN, has over 33,000 discharges annually [15]. Knox County, a county of 470, 313 people, is ranked 9th out of 95 counties in Tennessee for overall health [16]. Knox County has a higher than national average percentage of people with incomes below the poverty level [16]. In addition to Knoxville, UTMC also serves surrounding counties with lower health scores, lower income, and lower health literacy [17]. All of these factors contribute to lower health literacy, with low socioeconomic status being one of the main determinants of health literacy [18].

Although Preston Medical Library has offered a free health information service for over 25 years, there was a drastic increase in requests in 2014 due to the opening of the patient and family library inside of the hospital [19]. While there have been studies analyzing the health information service requests and the zip codes, there has not been a study comparing the library's top usage area in Knox County request topics with the hospital's reasons for hospitalizations and ED visits [20]. To examine the relationship between patron use of the library and a patient's use of the hospital, researchers partnered with the hospital's Performance Improvement Coordinator.

The first goal of this case study was to collaborate with the hospital to define the library's top usage area by zip code in Knox County. For this study, usage is defined as any use of the library, in person or otherwise, that resulted in health information being obtained by the patient. The second goal was to see why people were being hospitalized and if the reason for hospitalization aligned with the topics people were requesting health information on from the library. Lastly, the third goal was to evaluate the hospital's targeted zip codes for outreach to see if there was any overlap with the information service's top zip codes. The targeted zip codes for outreach were selected by the hospital as those with the greatest need based on their Community Needs Index Score [17]. Based on these results, outreach to community organizations was planned to reach the targeted zip codes with the lowest usage of the health information service.

## CASE PRESENTATION

IRB approval from the Graduate School of Medicine (IRB # 5169) was received. Researchers at the library requested data from the hospital by completing a Health Equity/Disparities Data Request Form to compare the health information service requests topics and zip codes from 2022 with the hospital's data from 2022. After completing the form, the health information service data was downloaded from the library's in-house database. Researchers de-identified the information, leaving only the date, zip code, city, method of contact, and customer text. Customer text was the health topic on which the person requested information. Researchers then coded the customer text to match the hospital's reasons for hospitalizations or Emergency Department (ED) visits, known as a “business line.” The hospital has defined 21 business line topics, or broad categorizations of reasons for hospitalizations or ED visits: Cardiac Surgery, Cardiology, ENT, General Medicine, General Surgery, Gynecology, Hematology/Oncology, Medical Oncology, Neonatology, Neurology, Neurosurgery, Obstetrics, Ophthalmology, Organ Transplant and Vent Assist Devices, Orthopedics, Psychiatry, Surgical Oncology, Thoracic Surgery, Trauma, Urology/Nephrology, and Vascular Surgery. Each customer text topic was given one business line topic that most closely matched. For example, if a person requested information on “allergies” or “diabetes,” that was given the General Medicine business line.

Data was sent to the hospital's Performance Improvement Coordinator to find the top five usage zip codes of the health information service. Furthermore, for each zip code in the top five, the coordinator found the business line and number of inquiries in each zip code for the health information service. Once that was completed, the coordinator pulled the hospital business line data for the top health information service zip codes. The information was then analyzed based on the business line topic to determine if the requests for information matched the reasons for hospitalization, called Inpatient, or reasons for ED usage.

The top zip codes that utilized the health information service in order of highest use to lowest were 37901, 37920, 37917, 37876, and 37725. The results were broken down and analyzed for each zip code. For zip code 37901, there was very low usage of the hospital, whether Inpatient or ED use. The most common use for Inpatient or ED was General Medicine; however, the library received more requests for information on Urology/Nephrology in that zip code. See Table 1 for the business lines and their usage related to Inpatient hospitalizations, ED visits, and library health information requests in 37901.

**Table 1:** Inpatient hospitalizations, ED visits, and Library Requests for patients associated with Zip code 37901.

Zip Code 37901			
Business line	Inpatient Hospitalizations	ED Visits	Library Requests
Cardiac Surgery	1	1	0
Cardiology	1	0	4
ENT	0	5	0
General Medicine	5	13	40
General Surgery	1	10	2
Gynecology	0	1	0
Medical Oncology	0	0	19
Neonatology	1	0	0
Neurology	1	0	3
Obstetrics	1	1	0
Ophthalmology	0	0	5
Psychiatry	0	1	0
Surgical Oncology	0	0	3
Urology/Nephrology	1	0	41

Zip code 37920 used the hospital the most for Inpatient and ED but was the second highest zip code that used the health information service. General Medicine was the most common use for Inpatient and ED and the most requested health information topic. For zip code 37917, the library's requests were mostly Neurology whereas the ED and Inpatient usage was largely General Medicine. See Table 2. Neurology had 69 Inpatients and the ED had 67 in zip code 37917, which is 5% and 2% versus 33% for the library's health information requests.

**Table 2:** Inpatient hospitalizations, ED visits, and Library Requests for patients associated with zip code 37917.

Zip Code 37917			
Business line	Inpatient Hospitalizations	ED Visits	Library Requests
Cardiac Surgery	45	325	0
Cardiology	72	16	1
ENT	8	262	0
General Medicine	431	1096	12
General Surgery	98	646	0
Gynecology	4	19	0
Hematology/Oncology	13	7	3
Medical Oncology	6	0	0
Neonatology	146	0	0
Neurology	69	64	15
Neurosurgery	17	0	0
Obstetrics	150	109	0
Ophthalmology	5	36	1
Organ Transplant and Vent Assist Devices	3	3	0
Orthopedics	98	44	4
Psychiatry	25	314	0
Surgical Oncology	7	0	0
Thoracic Surgery	8	0	0
Trauma	39	33	0
Urology/Nephrology	39	77	9
Vascular Surgery	9	0	0

The last two zip codes, 37876 and 37725, mainly requested health information on General Medicine, which matched the reasons for Inpatients and ED visits. In all zip codes, only 1% of health information requests were on Obstetrics / Gynecology, whereas Obstetrics / Gynecology comprised 14% of Inpatients and the ED in those five zip codes. Overall, in 2022, the topic people requested the most information on from the library was General Medicine, which aligned with the top reasons for hospitalizations and ED usage.

Researchers then compared the most used zip codes for the health information service with the hospital's targeted zip codes to determine if there was any overlap. The only zip code from the targeted zip codes in the library's top five was 37901. Therefore, researchers planned outreach for the other four zip codes: 37912, 37914, 37915, and 37921.

In researching possible organizations to contact to initiate outreach, it was decided that public libraries and local health departments would be the best places to contact [11]. Librarians contacted the public libraries in those zip codes and explained how the health information service could help them. They also offered to send brochures to the libraries to be displayed for their patrons. Two of the four libraries responded (zip codes 37912 and 37914) and were sent brochures.

In addition, researchers contacted health departments and health specific organizations in the four zip codes. Representatives from the health departments responded enthusiastically, and while no brochures were sent, a relationship was established between the library and the health departments. The administrative coordinator of one health department electronically forwarded information about the library's health information service to their colleagues and other areas in the organization. As a result, the library received communication from two separate individuals who had specific questions about the library's resources. The library sent information about mental health resources that will benefit the employees of the health department and their patients. These departments serve the public in all four zip codes, and each voiced their excitement over sharing the health information service with their populations.

### Discussion

The library's location in the middle of the hospital results in a symbiotic relationship that mutually benefits both, as health information is a valuable part of a hospital patient's care. The more information the library is able to provide for the hospital's patients, the more likely the care they receive in the hospital will result in positive outcomes [21]. Additionally, the more patients that come into the hospital results in a larger, more easily accessible population for the library to have contact with. By partnering with the hospital to identify zip codes, hospital usage, and information request topics, researchers were able to gather detailed information to determine if there was any overlap of zip codes and topics, which gave them a broader understanding of their population base. The overall takeaway from this project was that the most requested information from the library aligned with the top reasons for hospitalizations and ED usage.

Zip codes are one of the only consistent quantifiable pieces of data that the library records about the patrons who use the health information service, with many studies being published on the zip codes that use this service [22, 23]. By evaluating the area from which health requests come from, librarians can glean important information about their customers. Similarly, the hospital values the zip code data it records from its patients to evaluate its customer base and identify if they are reaching patients with the greatest need, as zip code impacts a patient's health and wellness [24]. The other big piece of information that both areas of service records is the health information need itself. By comparing these pieces of information, librarians determined that some patrons in these zip codes requested information that overlapped with why patrons from this same zip code are being hospitalized or going to the ED. Furthermore, the library and hospital's needs overlapped in that they both wanted to help patients in need and improve the patient experience.

When comparing the results, researchers found that three out of the five zip codes' top requests and hospitalizations or ED visits were for General Medicine. This is a potential weakness, as General Medicine is a broad topic and could reflect many different, non-related health conditions. It is also important to note that the library did not receive many information requests related to Obstetrics / Gynecology, whereas the hospital had many Inpatients and ED visits in this area. This might be because those patients with that business line are having a baby. However, there might be a need for the library to reach those patients in case they do need health information and are unaware of the service. In addition, it might be beneficial for the library to do outreach to the Obstetrics / Gynecology department at the hospital to make them aware of the resources the library provides [25].

Based on the results, the library targeted specific zip codes that the hospital determined were areas with the greatest need based on their Community Needs Index Score. While the original plan was to send brochures to community-based organizations in these zip codes, it became clear that electronic communication was also effective [26]. This information can be shared with the public, so while the library will receive no data when these transactions occur, these interactions may result in more community awareness about the library's resources.

Librarians will continue tracking where health information requests come from by recording patron's zip codes. This project will be considered successful if there is an increase of patrons from the targeted zip codes. In addition, the library has already received a health information request from a member of one of the health organizations that was contacted. Any measurable increase in presence in the targeted zip codes will be considered a valuable outcome from the marketing done in these areas. One challenge to this evaluation is that it depends on library staff recording the zip code for every patron who requests health information. While this is protocol, staff do not always remember to ask for the zip code. The second major issue is that awareness of the health information service may not result in people actually requesting health information. There is only so much that librarians can do to encourage people to use their services [27].

Hospital libraries may benefit from partnering with their hospital to show their impact on patient care. In addition, through the collection of hospital data, libraries can find which hospital service areas may need outreach to learn more about library services. Lastly, hospital libraries can evaluate if their consumers are asking health questions related to the top reasons for hospitalizations or ED visits.

Overall, this project strengthened the relationship between the hospital and the library through their work in analyzing zip code data and topic requests together. Furthermore, it led to librarian awareness of the hospital's targeted zip codes and provided a new goal to outreach to these areas. It has already led to new relationships between the library and local health departments, resulting in the library providing health information to organizations that have direct contact with the public.

## Data Availability

Data associated with this article cannot be made publicly available because they contain personally identifiable information. Access to the data can be requested from the corresponding author and may be subject to IRB restrictions.
